# The endosomal-lysosomal system: from acidification and cargo sorting to neurodegeneration

**DOI:** 10.1186/s40035-015-0041-1

**Published:** 2015-09-30

**Authors:** Yong-Bo Hu, Eric B Dammer, Ru-Jing Ren, Gang Wang

**Affiliations:** Department of Neurology & Neuroscience Institute, Ruijin Hospital affiliated to Shanghai Jiao Tong University School of Medicine, Shanghai, 200025 China; Department of Biochemistry, Center for Neurodegenerative Diseases, Emory University School of Medicine, Atlanta, GA 30322 USA

**Keywords:** The endosomal-lysosomal system, Neurodegeneration, Acidification, ESCRT, Retromer, Alzheimer’s disease

## Abstract

The endosomal-lysosomal system is made up of a set of intracellular membranous compartments that dynamically interconvert, which is comprised of early endosomes, recycling endosomes, late endosomes, and the lysosome. In addition, autophagosomes execute autophagy, which delivers intracellular contents to the lysosome. Maturation of endosomes and/or autophagosomes into a lysosome creates an unique acidic environment within the cell for proteolysis and recycling of unneeded cellular components into usable amino acids and other biomolecular building blocks. In the endocytic pathway, gradual maturation of endosomes into a lysosome and acidification of the late endosome are accompanied by vesicle trafficking, protein sorting and targeted degradation of some sorted cargo. Two opposing sorting systems are operating in these processes: the endosomal sorting complex required for transport (ESCRT) supports targeted degradation and the retromer supports retrograde retrieval of certain cargo. The endosomal-lysosomal system is emerging as a central player in a host of neurodegenerative diseases, demonstrating potential roles which are likely to be revealed in pathogenesis and for viable therapeutic strategies. Here we focus on the physiological process of endosomal-lysosomal maturation, acidification and sorting systems along the endocytic pathway, and further discuss relationships between abnormalities in the endosomal-lysosomal system and neurodegenerative diseases, especially Alzheimer’s disease (AD).

The endosomal-lysosomal system is a series of organelles in the endocytic pathway where various cargo molecules required for normal cellular function are internalized, recycled and modulated. Recently, mounting evidence has suggested that abnormalities in both endosomes and lysosomes, or dysregulation in their trafficking, play an important role directly in a surprising host of neurological dysfunctions, represented by AD, Parkinson’s disease (PD), and Lewy body dementia (LBD) [1–3]. Thus, the endosomal-lysosomal system is emerging as a key to understanding the mechanisms underlying both protein degradation and neurodegeneration. Here, we intend to summarize advances in the study of the endosomal-lysosomal system, with a focus on compartmentalized organization of trafficking routes, sorting machinery and their relationships to neurodegeneration.

## The endosomal-lysosomal system: a dynamic, interconnected vesicular network

Membrane dynamics which affect protein degradation and recycling within cells plays a critical role in maintaining homeostasis. Macromolecules and transmembrane proteins at the plasma membrane destined for degradation could enter the endosomal-lysosomal system via three broadly defined routes: endocytosis, autophagy or phagocytosis [4, 5]. Here we focus on the organization and functions of endocytic trafficking routes of protein/glycoprotein, and protein-bound lipid cargoes, preferentially over autophagy or phagocytosis.

The endocytic pathway is composed of a series of highly dynamic membrane-enclosed tubulo-vesicular structures. According to their different functions and roles in this system, several distinct types of compartments have been identified: early endosomes, recycling endosomes and late endosomes [6]. Additionally, lysosomes serve as organelles for storage of hydrolases, and are typically considered as a final destination where proteolytic degradation takes place. The membrane bilayer of endosomes and lysosomes creates an enclosed environment allowing an acidic pH, which is optimal for many hydrolases and other enzymes. Compared to a cytoplasmic pH (of about 7.0), the endosomal and lysosomal lumen pH is maintained in a range of 6.5 to 4.5, due to the activity of the ATP-dependent proton pumps present in the membrane of both endosomes and lysosomes [7]. Molecular trafficking and sorting along the endocytic pathway is regulated by the Rab family of small GTPases, which are critically important membrane association proteins. The Rabs function as molecular switches that alternate between two conformational states: the activated GTP-bound form and the GDP-bound inactivated form [8]. Different Rab proteins have corresponding host organelles; therefore, they are often regarded as markers of different endosomal compartments.

**Fig. 1:**
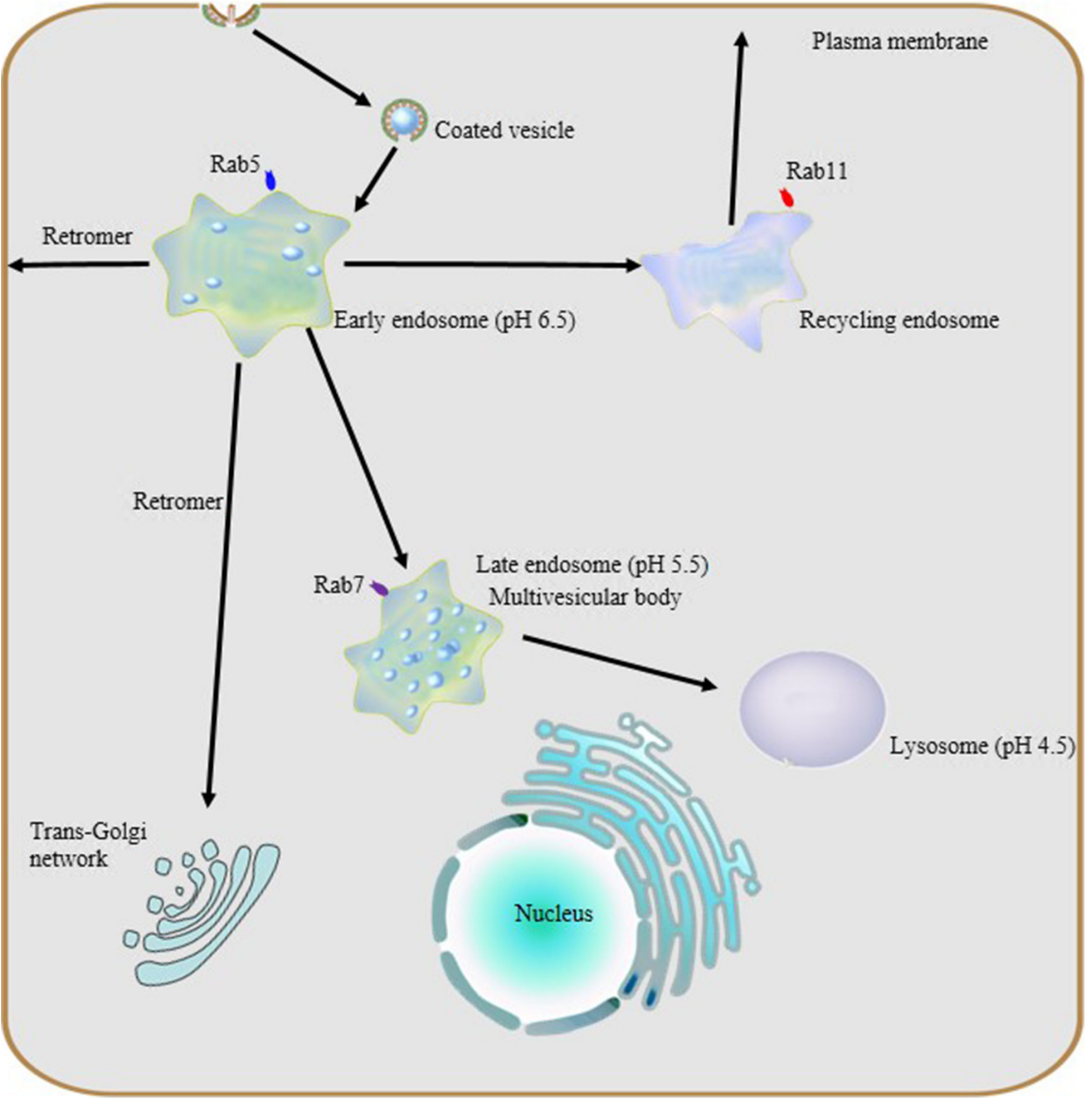
Endocytosis and endosome maturation. Protein internationalization is often dependent on a ubiquitous clathrin-mediated mechanism. Subsequent to internationalization, cargo proteins are transported to early endosomes via endocytic vesicles under the control of Rab5. Early endosomes serve as the major sorting stations where proteins can be sorted into recycling endosomes for recycling back to the cell surface, into a retrogradee pathway mediated by retromer to be sent back to the trans-golgi network (TGN), or into a degradation pathway for eventual targeting to the lysosome. As the number of intraluminal vesicles increases, early endosomes mature into late endosomes, and then late endosomes fuse with lysosomes.Due to their different capacities of acidification, a stable pH gradient is established in different compartments during the maturation process: early endosomes maintain pH at about 6.5, late endosomes at about 5.5 and lysosomes at about 4.5

Endosomes are dynamic, specialized compartments and undergo morphological and biological changes accompanied by vesicle trafficking. The maturation model states that endosomes along the endocytic pathway are transient and distinct compartments and, endosomes go through defined stages as they mature [6]. In this model, the process of endosomal maturation is characterized by four changes (Fig. 1): (1) an increasing number of intraluminal vesicles; (2) an increase in luminal acidification; (3) movement in space from the cell periphery towards the microtubule organizing center (MTOC); and (4) the switching of Rab proteins.

Initially, endocytosis (protein internalization) often starts at clathrin-coated pits that bud into vesicles derived from the plasma membrane. Within seconds after their formation, these vesicles can fuse with each other or with pre-existing early endosomes under the direction of the small GTPase Rab5 [9]. Early endosomes are generally formed in the peripheral cytoplasm with a slightly acidic intraluminal pH so that receptor cargo (ligands) can readily dissociate. The early endosome acts as a major sorting station, permitting the newly vacated receptors to recycle back to the cell surface for reuse, and directing dissociated ligands to be transported to late endosomes and finally to lysosomes for degradation. As mentioned, some membrane receptors along with membrane-bound lipids are transferred to recycling endosomes, returning to the plasma membrane, and this process is regulated by Rab4 and Rab11 [10, 11].

As internal vesicles bud inward from the membrane of the endosome, the number of intralumenal vesicles increases and this is an important transition in the maturation of early endosomes into late endosomes, and is the process whereby multivescular bodies (MVB) are created. The maturation from early to late endosomes is accompanied by the transition from association with Rab5 to Rab7, which is also known as “Rab conversion” [12]. Rab7 is a critical regulatory component that regulates transformation from early-to-late endosomes. In this process, endosomes move along a microtubule to the perinuclear region, regulated by interactions with dynein and kinesin and this process is accompanied by increasing intravacuolar acidification. The central role of late endosomes involves (1) the biogenesis of intralumenal vesicles and (2) they serve a role as a sink for sorting of ubiquitinated membrane proteins for eventual lysosomal degradation, both of which are executed by the ESCRT system of ubiquitin interacting and editing complexes [13]. At last, late endosomes or MVBs fuse with lysosomes and cargo proteins and intraluminal vesicles are degraded. It has been shown that late endosomes can be major sites of protein hydrolase activity acting on soluble proteins like ovalbumin taken up via earlier endocytosis, surpassing the activity found in lysosomes for these substrates, and so the entire endosomal-lysosomal compartment can be considered as a reservoir for hydrolases either active or inactive [14, 15]. Moreover, along the endosomal-lysosomal pathway, the concentration of acid hydrolases increases, while lumenal pH decreases, although activity varies by enzyme and not all are most active against all substrates in the lysosome [14]. In most neuronal cells, the pH of the lysosomal lumen can be as low as 4.5 [16]. Within this lumen, there are more than 50 hydrolases concentrated, and some of these show little to no sequence homology, suggesting convergent evolution. The fusion of late endosomes with lysosomes generally initiates the activation of hydrolase precursors or proenzymes, with a requirement for intraluminal Ca^2+^ and calmodulin [17], and prerequisite glycosylation with mannose-6-phosphate, which targets the hydrolases to receptors that concentrate them in the endosomal-lysosomal system [14]. Accordingly, lysosomes serve as the major site of activation of many proteolytic activities, even if their activity can be detected in other aspects of the endomembrane system.

The various stages of endocytosis with their branching decision tree for the sorting of receptors, receptor cargo, other transmembrane proteins, and hydrolase enzymes have a fundamental role in both membrane and protein processing, especially in neurons that require specialized regulation of endocytic and secretory pathways to ensure their normal function, particularly at synapses [18].

## A pH gradient established by vacuolar ATPase and chloride channels

### Vacuolar ATP-dependent acidification

As mentioned above, the endosomal and lysosomal compartments share at least one similar significant characteristic: low intraluminal pH. These sealed acidic compartments provide an appropriate environment for optimal degradation of substrate cargo and recycling of their cognate receptors. As macromolecules are transported along the endosomal-lysosomal pathway, the internal pH of both endosomes and lysosomes decreases rapidly due to vacuolar acidification. Previous research revealed that the same H^+^-ATPase, also known as vacuolar ATPase (V-ATPase), acidifies both endosomes and lysosomes. V-ATPase, differs from Na^+^,K^+^-ATPase in the plasma membrane, Ca^2+^-ATPase in the sarcoplasmic reticulum, and F_1_,F_0_-ATP synthase in mitochondria, in that it does not require a coupled influx of permeant anions [19]. Sulfhydryl alkylating reagents such as N-ethylmaleimide inhibit the V-ATPase dependent acidification of the endosomal-lysosomal system [19–21] as well as the specific inhibitor Bafilomycin A1 [22, 23]. V-ATPase is a unique class of ATPase present throughout the membranes which constrain the endocytic pathway, including the trans golgi network (TGN). V-ATPase, as a protein complex, is composed of two multimeric subunits, V_1_ in the cytoplasmic domain and V_0_ within the vacuolar membrane; the activity of V-ATPase depends on the dynamic assembly of these. V-ATPase is widely expressed in eukaryotic cells and serves as the master regulator of vesicular acidification in many subcellular membrane bound organelles. It also has important roles to play in vesicular trafficking and proteostasis.

### Chloride channel compensation

Since V-ATPase is electrogenic, continuous proton influx across membranes would result in an ever-increasing positive charge buildup in the lumen of endosomes and lysosomes. Since this potential difference would constitute an energetic barrier to maintenance of acidification, it is apparent that vacuoles must have a means of compensating. A series of studies have identified that chloride channels (CLC) conduct passive Cl^−^ ion current, which compensates for proton accumulation by V-ATPase [24, 25]. In mammals, CLC proteins form a large family of nine members and among them, CLC3 to CLC7 localize to membranes of the endosomal-lysosomal system. These vesicular CLCs are thought to stimulate V-ATPase activity in endosomal-lysosomal compartments, thereby facilitating acidification. To do this, CLCs permit H^+^ influx against a concentration gradient, but compensate by minimizing the electrochemical gradient, resulting in net regulation of the pH of vesicular compartments. In turn, CLC ion currents are regulated by the state of the intraluminal environment: for example, low lumenal pH is able to open CLC channel gating and Cl^−^ currents. Even though there are still controversies over the importance of CLCs, a number of studies have provided evidence that CLC defects in the nervous system of mice led to endosomal pH elevation, and dysfunctional cellular protein degradation, causing phenotypes very similar to human neurodegenerative diseases, like AD and PD [26, 27].

### Functions mediated by acidification

An acidic lumenal environment is necessary for proper function as follows [14]: (1) internalized receptors need acidic conditions in order to release their ligands and recycle back to the membrane; (2) the pH gradient along the endosomal-lysosomal pathway might direct the movement and maturation of these organelles [15]; (3) many hydrolases have optimal function at acidic pH; (4) acidic pH conditions may produce an environment favorable to oxidation reactions within the endosomal-lysosomal system.

## Two opposing cargo sorting systems: the retromer and ESCRT

On entering the endosomal-lysosomal network, internalized protein cargo has two potential destinies: either it is trafficked and delivered to the late endosomes and terminally, to lysosomes for degradation (via the ESCRT system); or, it is sorted and transported to the TGN or the plasma membrane for reuse (via the retromer).

### ESCRT: sorting ubiquitinated proteins for degradation

In the pathway promoting degradation, cargo proteins are transported into the intralumenal vesicles of endosomes for subsequent degradation. This process is initiated by a cargo-sorting receptor, namely ESCRT, such that target proteins are first captured in a signal-dependent manner. At the endosomal membrane, it is now evident that the ESCRT complexes serve to recognize and sort ubiquitinated endosomal proteins for degradation [13]. The machinery and molecular basis of this ubiquitin-dependent endosomal sorting is emerging through many studies in yeast as well as mammalian cells, some of which we highlight below.

The ESCRT machinery consists of four distinct protein complexes: ESCRT-0, I, II, and III, named for their order of recruitment and function in triggering ubiquitin-dependent sorting of cargo proteins into the MVBs of endosomes [28]. Advances in biochemistry and cell biology have provided a good picture of the architecture and interactions of the ESCRTs. The “conveyor belt model” for ESCRT complex function proposes that these complexes function sequentially to sort ubiquitinated proteins into MVBs, starting with ESCRT-0 and ending with ESCRT-III.

ESCRT-0, the least evolutionarily conserved of the ESCRT complexes, contains multiple ubiquitin-binding subunits or activities and is able to retain ubiquitylated proteins in or at the endosomal membrane, but its affinity for ubiquitin is very low. It is, however, able to support the clustering of ubiquitinated membrane receptors into patches of membrane, thereby increasing the local concentration of such receptors. Once ESCRT-0 has bound to an ubiquitinated protein, ESCRT-I, which has higher affinity, can take on the role of binding to the ubiquitinated substrate protein. ESCRT-I as well as ESCRT-II each have just one ubiquitin-binding domain, so that it is hypothesized they require ESCRT-0 for concentration of ubiquitin-tagged cargo prior to their binding [13]. ESCRT-III comprises several small, highly charged subunits, assembling in a highly ordered manner. The ESCRT-III complex has moonlighting roles in cytokinesis and viral budding, but is known to harbor deubiquitinase activity that acts to release ubiquitin from substrates successfully moving down the ESCRT conveyor. ESCRT-III also has functions in the genesis of MVBs by supporting invagination and pinching off of the endosomal membrane in regions that have sequestered cargo [29, 30].

### Retromer: a singular path for retrograde retrieval

Over the past couple of decades, there has been an increasing body of evidence implicating an ancient, evolutionarily conserved eukaryotic complex, which has come to be termed the “retromer.” The primary function of the retromer is to conduct multiple cargo-sorting events, mediating retrograde retrieval from endosomes to the TGN [31]. In yeast, the retromer is comprised of five subunits, all encoded by vacuolar protein sorting (VPS) genes: Vps35-Vps29-Vps26 are a trimeric core, while Vps5-Vps17 is a dimer of sorting nexin (SNX) proteins. Even though SNX proteins differ significantly between species, the trimeric protein is highly conserved, which is also named the cargo-selective complex (CSC) for its role in recognizing cargo proteins [32]. In this recycling pathway, the principal cargoes of retrieval are unliganded receptors that may have been missorted to lysosomes.

Even though there remain significant gaps in our understanding of specific mechanisms of retrograde trafficking, it is clear that, as pioneering studies have indicated, the retromer has cargo-specific recognition capacity, and that there are different forms of the complex acting on distinct sets of cargo through CSC association with different sorting nexins [33]. It is notable that the CSC trimer, despite its role as the core functional component of the retromer complex, has no membrane-binding activity and relies on nexins or Rab7 to execute its functions directed at membrane bound cargo. Previously, Vps35 was thought to provide the sole interface for cargo recognition, but recent studies have shown that other proteins such as Vps26, SNX3, and SNX27, are also implicated in cargo recognition in mammalian cells [31]. The sorting nexins recruit the CSC to endosomes and mediate its binding to the endosomal membrane. For example, SNX3, specifically recognizing phosphatidylinositol-3-monophosphate (which is enriched in early endosomes), can target the CSC to this compartment.

## Abnormalities and dysregulation of the endosomal-lysosomal system in neurodegeneration

**Fig. 2:**
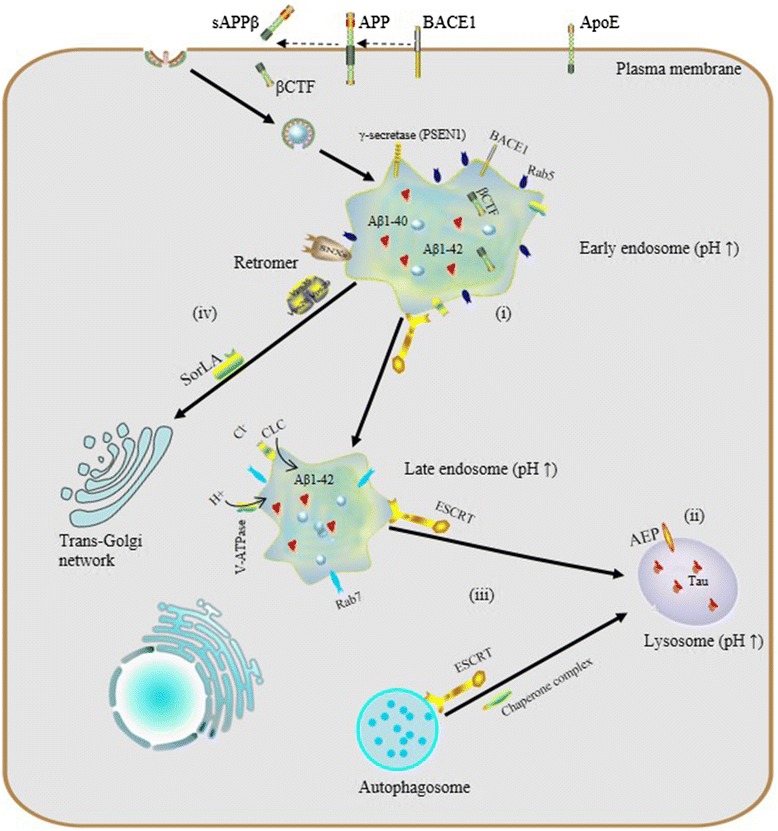
The endosomal–lysosomal system and AD. (i) endosomal enlargement, Rab5 overexpression and Aß accumulation; (ii) dysregulated endosomal-lysosomal acidification, pH elevation and tau aggregation; (iii) dysfunctional ESCRT complexes, defective autophagy and accumulation of intraluminal ubiquitinated proteins; (iv) defects of retromer: reduction of Vps26, Vps35 and SorLA disrupts the trafficking and processing of APP

Increasing attention being paid to the endosomal-lysosomal system has begun to elucidate a relationship between endosomal-lysosomal defects and neurodegeneration. In particular, robust pathology implicating endosomal-lysosomal disruption in AD has been well characterized. Here, we focus preferably on AD as a example of neurodegenerative disease and we believe that AD represents a general model of neurodegenerative diseases on abnormalities of the endosomal-lysosomal system occur along a continuum that includes early endosome changes, dysregulated acidification and sorting component defects (Fig. 2).

### “APP, in the early endosome, with beta secretase”: accomplices and bystanders

Even though many details remain to be resolved, a striking body of evidence indicates that disturbed vesicular trafficking has special relevance in AD and other neurodegenerative diseases [3]. In particular, as just described, early endosomes mark the location of initial amyloidogenic processing of APP by beta-site APP cleaving enzyme 1 (BACE1) and dissociation of ApoE from its receptor, LDLR1, upon internalization and the extent to which these events occur is dependent upon the time of residence in this compartment, thus attracting much attention. ApoE and its receptor genetically and functionally interact with APP, to influence its endocytosis and degradation [34, 35], thereby modulating the severity of amyloid pathogenesis.

Studies have demonstrated that BACE1 and APP proteolytic fragments, Aß and carboxyl-terminal APP fragment by BACE1 (ßCTF) are abundant in early endosomes [1, 36, 37]. For example, in cell models of AD pathology, the ßCTF has been found to be accumulated in early endosomes and it can disrupt neuronal ionic homeostasis in a concentraction-dependent manner [38]. Meanwhile, the dysfuction of BACE1 retrograding from the endosomes into the TGN, appears to enhance the production of ßCTF, leading to an increase of Aß in early endosomes. In the brains of AD patients, Cataldo et al. found that, even at earliest stage of disease when neurons exhibit enlarged Rab5-positive endosomes containning Aß immnoreactivity [39]. Moreover, Aß1-42 may also accumulate in lysosomes, disrupting the lysosomal membrane [40]. As a consequence, it has been noted that ectopic, elevated levels of lysosomal hydrolases can be observed in the CSF of individuals with AD [41]. It was first reported almost two decades ago that in familial AD, Rab5 is overexpressed or stabilized post-translationally concommitant with a very marked increase in pyramidal neuron early endosome volume, and this results in increased processing of APP, a sorting imbalance, and decreases in neurotrophin receptor expression [42, 43]. Enlargement of the early endosome compartment has been considered as the earliest pathological change in AD, even occurring decades before clinical symptoms are evident [44]. Further, swollen endosomal profiles have been postulated to represent activation of the endosomal-lysosomal pathway, involving increased rates of endocytosis and vesicular turnover. Consistent with this, studies using Rab5 as a specific endosomal marker have identified a relationship between increased endocytosis and an enlargement in endosome size and volume [45]. Interestingly, a number of Rabs that regulate the flux of traffic through early endosomes directly or in adjacent compartments have been found by RNAi screen to be either positive or negative regulators of Aß production in heterologous mutant APP expressing cell lines [46]. Another recent review that delves into effects of interactors in regulating traffic of APP and secretases, particularly considering events increasing their co-location throughout the endomembrane system as opportunities for alternative APP processing events [47].

Even though it is established that AD is a heterogeneous disease, perturbation of the neuronal endosomal-lysosomal pathway is one cellular feature shared in common by all subtypes of AD. Therefore, it is tempting to speculate that cellular pathological changes affecting this system are early and essential initiating events in AD pathogenesis, regardless of disease subtype and genetic predisposition.

### Dysregulated acidification, cellular indigestion?

Endosomal-lysosomal pH defects are an emerging theme in mechanisms underlying a number of neurodegenerative diseases. To date, results from experiments *in vivo* and *in vitro* have revealed the importance of proper vesicular pH balance and optimal acidification in transporting and degrading cargo via the endocytic pathway [48, 49]. For instance, Lee et al. reported that, in presenilin1 (PSEN1)-deleted blastocysts, defective lysosome acidification was observed with a substaintially elevated lysosomal PH of 5.4 and PSEN1 is essential for the transport of mature V0a1 subunites of V-ATP to lysosomes for their acidification and proteolysis [50]. Specifically, dysregulation of acidification and intracellular pH perturbation could influence the activity of enzymes in endomembrane compartments, resulting in impaired clearance of protein aggregates downstream of elevated endomembrane system pH, or conversely, due to decreased cytoplasmic pH. Regarding the latter, asparaginyl endopeptidase (AEP) is a typical pH-sensitive protein hydrolase the activity of which depends on the acidic pH of vesicular compartments. Predominantly localized in late endosomes, asparaginyl endopeptidase (AEP) specifically cleaves substrates with an asparagine residue at the P1 site. It is known that AEP can undergo reversible pH-dependent autoproteolytic activation, and in normal conditions, full-length pro-AEP is inactive [51]. As pH decreases from neutral to acidic, the activity of AEP gradually increases, such that it is partially activated at pH 4.5 and fully activated at pH 3.5, via removal of a cap that covers the active site. In AD patients, lysosomal acidification may be defective and it has been shown that the intracellular pH of neurons gradually decreases with aging [52] and more so with lactic acid elevation seen in AD cortex [53], so ectopic AEP activation or activity after leakage of active enzyme from late endosomes or lysosomes may be increased. AEP is involved in pathological tau degradation. Specifically, AEP generates tau fragments that form insoluble fibrils and result in neurotoxicity and neuropathological changes in AD [54, 55].

Increased endosome and lysosome pH is expected to have global effects on the proteome, particularly membrane proteins which rely on this pathway for their regulation and degradation. Interestingly, studies of microglia in culture have shown that in the absence of inflammatory IL-6 signaling, microglia do not achieve a sufficiently low lysosomal pH to degrade Aß, while after stimulation, CLC7 trafficking to lysosomes increases and pH drops sufficiently to improve Aß clearance [49, 56]. We have recently performed a systematic look at the proteomic effects of defective endosomal-lysosomal pH in a cellular model, in order to develop a better understanding of the global changes in the proteome that follow inhibition of V-ATPase and could be considered together as a signature or biomarker of defective vacuolar acidification [57, 58], which would be expected to have an overlap with changes seen in AD and/or other conditions which may be subject to this often age-dependent defect. Indeed, blocking lysosomal degradation with bafilomycin A1 affects a significant increase in global K63 polyubiquitin linkages, which also occurs in AD, but AD brain global ubiquitin linkage profiling shows changes in other linkages as well [59]. Since K63 linked ubiquitin is not targeted to the proteasome, but does increase with V-ATPase acidification in the model of lysosomal insufficiency, the increase in K63 linkages seen in AD implicates accumulation of ubiquitinated proteins with obligate ESCRT-mediated degradation. Thus, trafficking, inflammatory signaling, and cell-type specific roles of dynamic lysosomal acidification are becoming increasingly appreciated for potential roles in AD pathogenesis.

### Roles of sorting components in AD

#### The ESCRTs

A number of neurodegenerative diseases, including AD, are characterized by accumulation of intracellular ubiquitinated proteins which can be actively collected into aggregates that are usually targeted to autophagosomes, thus implicating defective autophagy. The ESCRT complexes are involved in multiple cellular processes, including efficient fusion of autophagic vesicles for bulk degradation of cargo proteins. Autophagy serves as an intracellular clearance mechanism, preventing the accumulation of proteins that disrupt neuronal function and eventually lead to neurodegeneration [60, 61].

Interestingly, in early stages of AD, autophagy in neurons is activated, but becomes compromised as the disease progresses. There are more and more studies to identify the signal molecular machinery involving autophagy and the ESCRTs. Studies indicate that the ESCRT machinery is not only involved in sorting ubiquitinated cargo but also in initiating vesicle formation within endosomes becoming MVBs [62]. The most direct evidence for an ESCRT role in protection of neurons from protein accumulation and neurodegeneration has been seen upon overexpression of charged multivesicular body protein 2B (CHMP2B), which is a subunit of the ESCRT-III complex. CHMP2B overexpression in neurons impairs lysosomal degradation of internalized proteins, resulting in accumulation of autophagosome, dendritic retraction and neuronal loss [63]. Typically, the autophagic pathway converges with the endocytic pathway at a point where mature autophagosomes fuse with MVBs. Compromised ESCRT function blocks the maturation and the proper turnover of autophagosomes, while functional ESCRT complexes are required for autophagic fusion and efficient degradation [64, 65].

#### The retromer

Over the years, researchers have studied neurodegenerative diseases arising from defects of retromer sorting proteins [66–68]. Particular interest has been taken in the link between retromer function to AD pathogenesis [69]. First, it was proposed that the retromer mediates endosome-to-TGN traffic of beta secretase, particularly BACE1, a transmembrane protein and enzyme that cleaves APP, promoting amyloidogenic and neurotoxic release of the ßCTF that is the precursor of Aß species, as well as the C-terminal fragment APP intracellular domain (AICD), and this event is considered to be upstream of neurotoxic effects resulting from BACE1 activity [70]. Dysfunction of the retromer increases the concentration of BACE1 in the endosomal compartments, thereby providing more of an opportunity for the cleavage of APP, Aß production and ultimately,extracellular Aß-containing plaques [66, 71]. In the brains of patients with AD, expression of Vps26 and Vps35 is reduced. Since these are two critical components of the retromer CSC trimer that recognize retromer cargo including BACE1, loss of function for the CSC would be expected to promote amyloidogenesis. Indeed, another study found that Vps35 mutation leads to up-regulation of Aß generation in mouse models [72]. Stabilizing the retromer complex via a drug-like chemical chaperone was found to decrease Aß production and increase neuroprotective alpha cleavage, probably by shifting APP out of early endosomes and improving functional retromer complex levels [73].

Interestingly, the above mentioned chemical chaperone also shifted the localization of SorLA, which associates with both Vps26 and APP, modulating amyloidogenic cleavage of APP [74] by upregulating the sorting of APP into endosomal compartments [75]. Recently, a comprehensive study proved that reduced SorLA expression is associated with mild cognitive impairment (MCI) and AD [76]. In cultured hippocampal neurons, studies have confirmed important roles of SorLA in trafficking and processing of APP. SorLA direct Aß peptides to late endosomes and lysosomes, consequently, promoting degradation and clearance [77, 78].

Finally, it is important to point out that the general processes of endocytosis and endosomal-lysosomal dysregulations above-mentioned, have profoundly distinct implications for potential functions associated with other neurodegenerative diseases, such as PD, ALS, and Frontotemporal lobe degeneration (FTLD).

## Conclusions and perspective

The endosomal-lysosomal system is a complex and highly dynamic process, where internalized transmembrane proteins, receptors, receptor ligands, and some soluble extracellular proteins are transported, sorted, and/or degraded. In recent years, particular attention has been paid to the endosomal-lysosomal system because it is involved in almost all of the neurodegenerative diseases, even though how it does so in each still remains unclear. Ongoing future studies will investigate both common and cell-type (or even local membrane region) specific trafficking and proteostasis pathways involving the endosomal-lysosomal system as well as the larger endomembrane system. For example, a better understanding of distinct roles that ubiquitination plays in ESCRT-mediated proteostasis (and even lipid droplet homeostasis [79] which appears to be dysregulated in glia in neurodegeneration [47, 80]) could help to predict and ultimately therapeutically address the onset and progression of neurodegenerative diseases for specific individuals or sub-populations. This milieu of membrane-bound proteins that dynamically sorts cargo enriched for signaling, inflammation, and neurotrophic functions—among others—promises to provide a mother lode of new therapeutic targets for amelioriating neurodegenerative diseases, but the exploration also promises to be challenging, requiring the development of novel techniques and insight.
